# An optimised saliva collection method to produce high-yield, high-quality RNA for translational research

**DOI:** 10.1371/journal.pone.0229791

**Published:** 2020-03-09

**Authors:** Roisin Sullivan, Susan Heavey, David G. Graham, Rachel Wellman, Saif Khan, Sri Thrumurthy, Benjamin S. Simpson, Tina Baker, Sarah Jevons, Jose Ariza, Victor Eneh, Hayley Pye, Hayley Luxton, Rifat Hamoudi, Hayley Whitaker, Laurence B. Lovat

**Affiliations:** 1 Gastroenterological Intervention Centre (GENIE) and Molecular Diagnostics and Therapeutics Group, University College London, London, England, United Kingdom; 2 Molecular Diagnostics and Therapeutics Group, Division of Surgery & Interventional Science, University College London, London, England, United Kingdom; 3 University College London Hospital, London, England, United Kingdom; 4 Department of Clinical Sciences, Sharjah Institute of Medical Research, College of Medicine, University of Sharjah, Sharjah, United Arab Emirates; University of Helsinki, FINLAND

## Abstract

Saliva represents an ideal matrix for diagnostic biomarker development as it is readily available and requires no invasive collection procedures. However, salivary RNA is labile and rapidly degrades. Previous attempts to isolate RNA from saliva have yielded poor quality and low concentrations. Here we compare collection and processing methods and propose an approach for future studies. The effects of RNA stabilisers, storage temperatures, length of storage and fasting windows were investigated on pooled saliva samples from healthy volunteers. Isolated RNA was assessed for concentration and quality. Bacterial growth was investigated through RT-PCR using bacterial and human primers. Optimal conditions were implemented and quality controlled in a clinical setting. The addition of RNA*later* increased mean RNA yield from 4912 ng/μl to 15,473 ng and RNA Integrity Number (RIN) from 4.5 to 7.0. No significant changes to RNA yield were observed for storage at room temperature beyond 1 day or at -80 °C. Bacterial growth did not occur in samples stored at ambient temperature for up to a week. There was a trend towards higher RNA concentration when saliva was collected after overnight fasting but no effect on RIN. In the clinic, RNA yields of 6307 ng and RINs of 3.9 were achieved, improving on previous reports. The method we describe here is a robust, clinically feasible saliva collection method using preservative that gives high concentrations and improved RINs compared to saliva collected without preservative.

## Introduction

Use of saliva as a liquid biopsy has recently increased, providing a real-time measure of a patient’s disease state and enabling a wide-range of translational applications that spans medicine, dentistry, microbiology and forensic science [1–9]. It is also widely used for exploring the transcriptome, proteome, metabolome and even inflammatory and stress responses of animals such as canines, non-human primates, pigs and sandflies [10–14]. It is an easily accessible biofluid or matrix that offers a non-invasive collection technique, reducing the discomfort and distress associated with blood and urine collection. Laidi et al., 2014 used salivary and serum tumour marker CA 15–3 in healthy woman and breast cancer patients to show a significant positive correlation between saliva and serum, suggesting it could be used as an alternative to blood sampling [1].

Despite the notable advantages of using saliva several challenges have slowed the development of salivary liquid biopsy. The constituents of saliva vary significantly both within and between individuals subject to collection method and rate of salivary flow, which ranges from 1–1.5L daily [15]. Hydration and circadian rhythms also significantly affect salivary flow rate [15,16]. In addition, the human oral microbiome contains over 600 prokaryotic species within the oral cavity with up to 10^8^ microorganisms per millilitre of saliva [17–19].

Informative analytes appear in lower concentrations in saliva than blood, potentially affecting downstream applications, although recent advances in both proteomic and transcriptomic technologies have allowed the screening of saliva at a much higher sensitivity [20–22]. RNA is extremely labile and sensitive to rapid degradation leading to alterations in expression profiles, with endonucleases found to primarily contribute to the partial degradation of salivary RNA [23]. Interestingly cancer patients have been reported to have elevated RNase activity which could hamper the use of saliva as a liquid biopsy [24]. However, the transcriptome has been reported to be stable enough in saliva to detect a large panel of human mRNA using microarray technology, highlighting the potential utility of salivary transcriptomics, and the importance of high-quality collection and processing procedures [25].

Existing saliva collection methods include unstimulated or resting saliva collection via passive drooling, scraping the buccal mucosa or direct aspiration from the floor of the mouth [1,16,25,2,3,4,26,27].

Stimulated salivary collection uses masticatory or gustatory stimulus, including the direct application of 2% citric acid onto the tongue [2]. Previous salivary studies have also employed mouth rinsing prior to collection, 1.5 hour fasting periods, and storage at -80 °C with or without centrifugation [1,19,25,3,4,26]. These recommendations may not always be feasible in a clinical setting and reduce the practicality of at-home saliva sampling.

Previously, work by Pandit et al., 2013 on saliva RNA- extraction methods suggested that collection under RNAase-free conditions or addition of RNA stabilizers was not required [27]. However, as whole blood can be routinely collected with the addition of commercially available preservation buffers that contain RNA stabilizing agents including RNA*later*, RNAprotect, PAXgene and TRIzol we felt that this required further investigation. Previously, concerns have been raised about potential genomic DNA (gDNA) contamination associated with RNA stabilizer use in salivary desquamated oral epithelial cells, however RNA*later* was found to produce higher quality RNA than available alternatives [28,29].

The aim of this study is to establish a standardised and reproducible salivary collection method to produce high-quality RNA to improve the performance of downstream translational applications. Here we investigate factors within the collection process which could affect the quality of salivary RNA such as storage at ambient temperatures, fasting windows prior to saliva collection, and collecting saliva with and without the addition of a preservative.

## Materials and methods

### Healthy volunteer cohort

Nine healthy volunteers were consented under the ethically approved ‘Saliva To Predict Risk of Disease Using Transcriptomics and Epigenetics (SPIT) study (ISRCTN Registration: 11921553) over a three month period between June 2016 and August 2016. Recruitment was carried out using written consent and ethical approval. The same volunteers were used throughout each experiment, with three volunteers making up each replicate pool. The cohort was comprised of 6 females and 3 males, with ages ranging from 18–47 years, and a median age of 30.5 years.

### Clinical cohort

Saliva was collected as part of the ethically approved SPIT study (ISRCTN Registration: 11921553) and from University College Hospital London (UCLH) using patients participating in the ‘Barrett’s Oesophagus surveillance with Optical biopsy using Spectroscopy and enhanced endoscopic imaging to Target high risk lesions’ (BOOST) clinical trial (ISRCTN Registration: 58235785) following approval from the NHS National Research Ethics Service Committee London (Dulwich). Recruitment occurred between June 2008 and March 2017. All patients who were unfit to undergo endoscopy, unable to give informed consent, pregnant, under the age of 21 and non-English speakers were excluded. Samples represented a small subset of a much larger population and were chosen for heterogeneity. Recruitment was carried out using written consent and with ethical approval. All research was performed in accordance with relevant guidelines/regulations and all participants provided informed consent. Participants were patients undergoing clinical endoscopy for Barrett’s Oesophagus (BE) assessment or treatment (n = 65). The cohort consisted of 15 females, 50 males with ages ranging from 40–89 years, with a median age of 66 years. All volunteers gave informed consent.

### Comparison of different nucleotide extraction methods

Saliva was collected from four healthy volunteers who each gave 3 x 1 ml samples which were each mixed with 1 ml RNA*later* (Sigma-Aldrich, cat no. R0901). 1 ml of the saliva and RNA*later* mix from each volunteer was mixed into three separate pool replicates, which were then centrifuged at 1000 rpm for ten minutes to remove cell debris. Four different extraction kits were compared; Qiagen DNA/RNA AllPrep Mini Kit (cat no. 80204), Zymo RNA Clean and Concentrator-25 (cat no. R1017) (following a traditional phenol-chloroform extraction), Zymo DirectZol Miniprep Plus kit (cat no. R2051) and Zymo Quick Prep RNA (cat no. R1050). 800 μl of sample and preservative mix from each pool was extracted using each of the four kits. Manufacturer’s protocols were followed except for Zymo DirectZol Miniprep Plus kit, where a 6:1 ratio of QIAzol (Qiagen, cat no. 79306) to sample was used instead of 10:1.

### RNA extraction

All RNA extractions were performed using the Zymo DirectZol RNA Miniprep Plus Kit (cat no. R2051) according to manufacturer’s instructions. 800μl of the saliva alone or saliva and preservative mix was treated with 4.8 ml of QIAzol (Qiagen) and the eluent passed through the column once more. RNA samples were eluted in 40 μl nuclease-free water and after quality control, all samples were stored at -80 °C.

### RNA quality control

The RNA concentration (ng/μl) and 260/230nm and 260/280nm ratios were analysed using the DeNovix DS-11 FX + Spectrophotometer/Fluorometer. RNA integrity (RIN) was analysed using the Agilent Bioanalyzer and RNA 6000 Nano Chip Kit, and the Eukaryote Total RNA v2.6 assay software.

### Turbo DNase treatment

All UCLH patient samples were treated with Turbo DNAse using the Ambion Turbo DNA*free* kit (Thermo Fisher, cat no. AM1907) immediately after extraction as per the manufacturer’s protocol. Samples were then stored at -80 °C.

### Sample collection for preservative comparison

**Fig 1:**
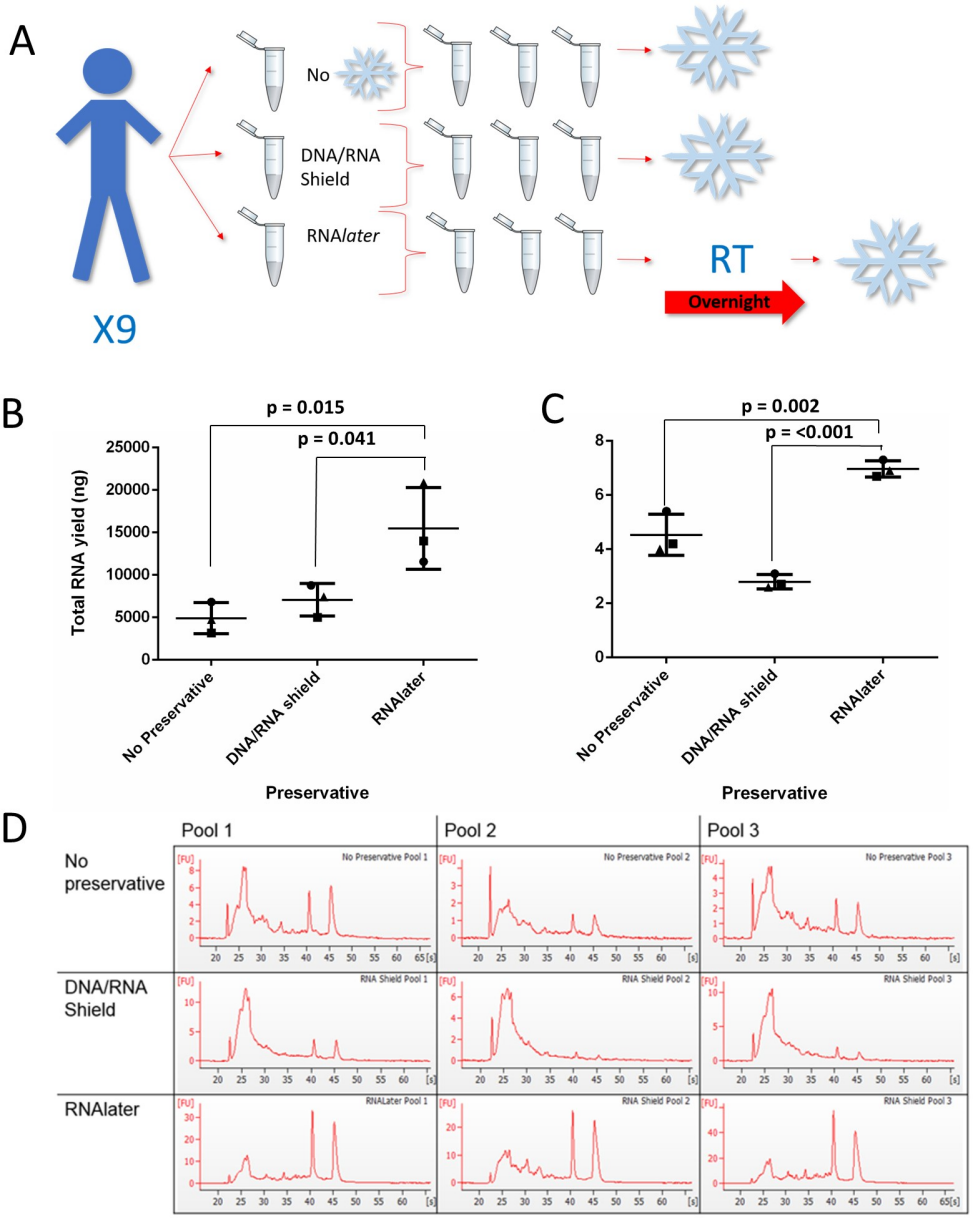
The effect of saliva collection with preservatives on RNA yield and quality. To assess the effect of preservatives, saliva samples were collected from nine volunteers with two different preservatives and without and were pooled into three. (**A**) An overview of the experimental protocol showing how collection with the different preservatives was performed. Snowflake symbols indicate freezing at -80°C and RT = room temperature storage. (**B**) The yield was assessed using spectrophotometry (ng). (**C**) RNA integrity was assessed through a RIN value for each pool. A one-way ANOVA and a Tukey test were performed. The three different pools of samples are represented as circles, squares or triangles. Bars represent the mean concentration of the three pools while error bars represent SD. (**D**) Bioanalyzer traces for each pool of samples and each preservative are shown. Artwork was either drawn by the authors or downloaded from https://openclipart.org under the creative commons license.

As set out in Fig 1A, each of nine healthy, overnight-fasted volunteers were given three pre-prepared 50 ml Falcon tubes containing either 1 ml of DNA/RNA Shield (Zymo, cat no. R1100-50), 1 ml of RNA*later* or an empty tube (no preservative). Volunteers were allocated randomised orders of donation of saliva to prevent bias. Each volunteer donated 1 ml of unstimulated whole saliva via passive drool per tube, agitating tubes containing preservative throughout collection. The tubes containing no preservative were immediately placed on ice. Samples containing preservatives were left at ambient temperatures for 1 hour. Three pools for each preservative condition were created, each containing saliva from three volunteers, to reduce variability between samples. Each pool was were then centrifuged at 1000 rpm for ten minutes to remove cell debris. The pools containing no preservative and DNA/RNA shield were immediately stored at -80°C. The pool containing RNA*later* was left at room temperature overnight before moving to -80°C storage the following day in accordance with the manufacturer’s recommendations. This method of saliva collection was used for all following experiments with notable alterations described below.

### Storage temperature study and bacterial load study sample collection

For these analyses, saliva samples were collected as described in the ‘Sample collection for preservative comparison’ method using RNA*later*. Three pools of saliva were created from 9 volunteers as replicates and initially stored at room temperature. 800 μl of the saliva and preservative mix was taken in the morning from each pool on each day for seven days, centrifuged at 1000 rpm for ten minutes and aliquots stored at -80°C immediately.

### Saliva stability after storage at -80°C

1ml saliva samples were collected from 9 volunteers using the method described in the ‘Sample collection for preservative comparison method’ using RNA*later* and stored at room temperature for 24 hours before pooling to create the three replicate sample pools. Each pool was were then centrifuged at 1000 rpm for ten minutes to remove cell debris. Four aliquots of 800 μl of saliva and preservative mix was taken from each pool and immediately stored at -80 °C, then extracted after 1, 2, 3 and 4 weeks.

### Cell culture

OE33 cells, sourced from the European Collection of Authenticated Cell Cultures (ECACC) (Cat no. 96070808) and mycoplasma tested, were maintained in RPMI -1640 (Sigma Life Sciences) supplemented with 10% foetal bovine serum (FBS; Life Technologies) and 5ml L-glutamine (GIBCO Life Technologies). Cells were passaged every three days at 80% confluence. RNA was extracted from OE33 cells at passage 10, using the DirectZol MiniPrep Kit (Zymo) and stored at -80 °C until required.

### Bacterial culture

DH5-α bacteria (Thermo Fisher Scientific) were cultured in LB broth over 8 hours in a shaking incubator (37 °C, 3200 rpm). The media was centrifuged at 4 °C for 20 mins at 3200 rpm and cell pellet stored at 20 °C. RNA was extracted using DirectZol Miniprep Kit (Zymo) using manufacturer’s protocol and stored at -80°C until required.

### qRT-PCR

cDNA was synthesised using 0.5 μg of RNA from all samples using iTaq universal SYBR Green Supermix (BIO-RAD, cat no. 1725120) and the High Capacity cDNA Reverse Transcription Kit (Applies Biosystems, cat no. 4368814). Bacterial DNA was analysed via RT-PCR, using primers that recognised bacterial 16S (forward (8F: 5’AGAGTTTGATCCTGGCTCAG-3’ which targers universally, reverse (336R): 5’-ACTGCTGCSYCCCGTAGGAGTCT-3 targeting conserved positions 358–336 in 16s rRNA ‘) (Sigma Aldrich) and 18S (forward: 5’- TGACTCAACACGGGAAAC-3’, reverse: 5’TCGCTCCACCAACTAAGAAC-3’) primers (Eurofin) that were validated using melt and standard curves based on control cDNA from OE33 or DH5-α. Raw cycle threshold (Ct) scores were gathered for both 18S and 16S, with Ct scores being inversely proportional to expression.

### Fasting study saliva collection

Nine healthy volunteers fasted overnight and then provided 1 ml of saliva upon waking which was collected into 1 ml of RNA*later*. All volunteers then ate and drank and rinsed their mouths with water before donating their first post-prandial saliva sample. They then fasted until the end of the study. All collections were made in the morning at 1, 2, 3 and 4 hours after eating. Volunteers were allowed a few sips of water after each donation. All samples were stored at room temperature for 24 hours before three sample pools were created for each collection timepoint, each containing the same three volunteers for each pool to minimise variation due to inter-volunteer differences. Each pool was were then centrifuged at 1000 rpm for ten minutes to remove cell and food debris. 800 μl of saliva and preservative mix was taken from each pool and stored at 80 °C prior to extraction.

### Clinical validation: Sample collection from patients

1 ml of saliva was collected from fasted patients prior to endoscopy procedure in a pre-prepared tube containing 1 ml of RNA*later* which was inverted 4–5 times, left overnight at room temperature, centrifuged at 1000 rpm for ten minutes and then stored at -80 °C in 800 μl aliquots. Patients who were being assessed for oesophageal cancer at endoscopy were categorised into risk profiles, from low through to high risk. These included, in order of increasing risk, those without Barrett’s oesophagus who were labelled as normal (n = 17), non-dysplastic Barrett’s oesophagus (n = 22), dysplastic Barrett’s oesophagus (n = 12), and oesophageal adenocarcinoma (n = 11). All fasted for a minimum of 6 hours prior to saliva collection, as required for endoscopy. It should be noted that not all patients were able to produce 1 ml of saliva, due to their ill-health, previous 6 hour fasting window and time-constraints before their endoscopy.

### Data analysis

One-way ANOVA (unpaired analyses) with Tukey post-test statistical tests were generated using GraphPad Prism software, with p < 0.05 deemed statistically significant. All averages are shown as the mean ±the standard deviation.

## Results

### RNA*later* preservative stabilises salivary nucleotides

A comparison of different nucleotide extraction kits using pooled saliva showed that the Zymo Direct-Zol RNA Miniprep Plus Kit gave the highest RNA yield without compromising sample quality (Table 1). To investigate if quality and/or concentration and yield of RNA isolated from saliva could be improved with the addition of commonly available preservatives, saliva was collected either without preservative or in the presence of the preservatives RNA*later* or DNA/RNA Shield (Fig 1A). All RNA yields given (ng) refer to per mL of saliva only. RNA*later* had a total RNA yield of 15473.2 ng (± 4824.7), in comparison to 7073.2 ng (±1916.3, p = 0.041) for DNA/RNA Shield and 4911.7 ng (±1824.1, p = 0.015) for saliva collected without preservative.

**Table 1:** Nucleotide extraction kit comparison.

	RIN	260:280	260:230	Concentration(Nanodrop, ng/μl)	Concentration (Bioanalyzer ng/μl)
**Qiagen Allprep**	8.4	1.34	2.06	44.55	116
**Zymo Clean and Concentrate 25**	7.2	1.95	1.58	120.73	161
**Zymo Directzol**	7.5	2.00	1.80	176.09	339
**Zymo Quick Prep RNA**	7.7	1.99	2.28	53.61	49

Details of different nucleotide extraction kits tested on pooled salvia with details of quality controls including concentrations, RIN and A260/280 and A260/230 ratios.

RNA*later* also produced an RNA concentration of 386.83 ng/μl (± 120.62), in comparison to 176.83 ng/μl (±47.91, p = 0.041) for DNA/RNA Shield and 122.79 ng/μl (±45.6, p = 0.015) for saliva collected without preservative (S2A Fig). To assess RNA quality, samples were assessed using the bioanalyzer, where intact 18S and 28S peaks with no degraded RNA in the inter region would be noted as having a maximum RIN of 10. Saliva collected with RNA*later* gave the highest mean RIN of 7.0 (±0.3), significantly higher than both saliva collected with DNA/RNA Shield (2.8, ±0.26, p = <0.0001) and saliva collected without preservative (4.5, ±0.75, p = 0.002) (Fig 1C and 1D). As RNA*later* gave the best results all additional experiments were completed with RNA*later* only.

### Room temperature storage does not adversely affect nucleotides

**Fig 2:**
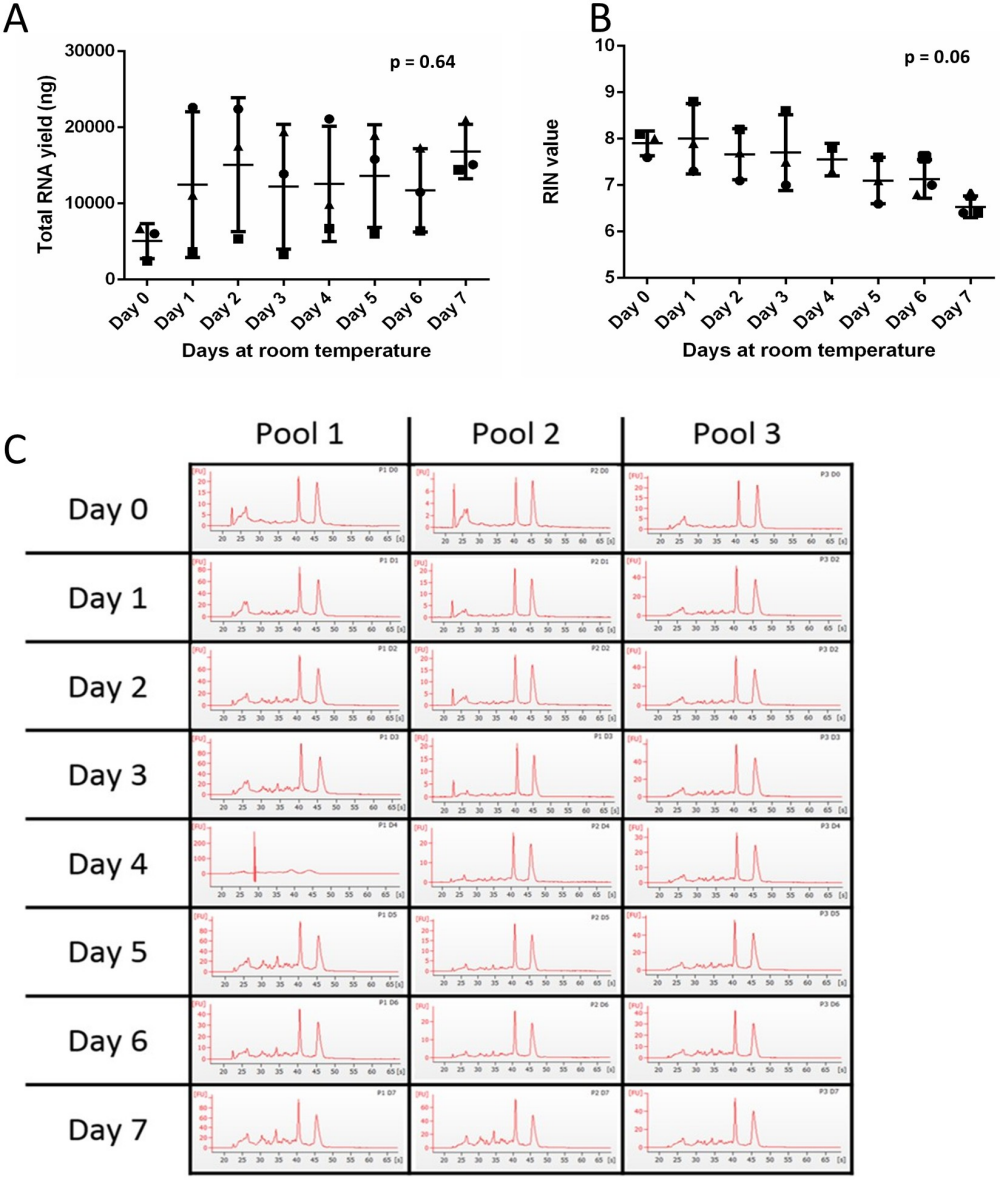
Effects of ambient storage lengths on quality and yield of salivary RNA. Three pools of saliva samples were collected in RNA*later* from nine volunteers. Each pool was stored at ambient temperature, with saliva taken and stored at -80 ° C on days 0, 1, 2, 3, 4, 5, 6, and 7. (**A & B)** RNA yield (ng) and RIN value were assessed. A one-way ANOVA and Tukey test were performed. The three different pools of samples are represented as circles, squares or triangles. The error bars represent SD. (**C**) Bioanalyzer traces for each pool at each timepoint are shown.

According to the manufacturer, saliva preserved in RNA*later* should be stored at room temperature for one day before freezing. To assess the effects of this, samples were incubated at room temperature for up to seven days, with 800 μl of sample stored at -80 °C on days 0, 1, 2, 3, 4, 5, 6, and 7. There was no significant change in either RIN or RNA yield or concentration in samples incubated at room temperature for longer than 24 hours although there was an increase in RNA concentration from day 0 to day 1 in two out of the three pools (Fig 2 and S2B Fig).

### Bacterial growth at ambient temperature is not significant

**Fig 3:**
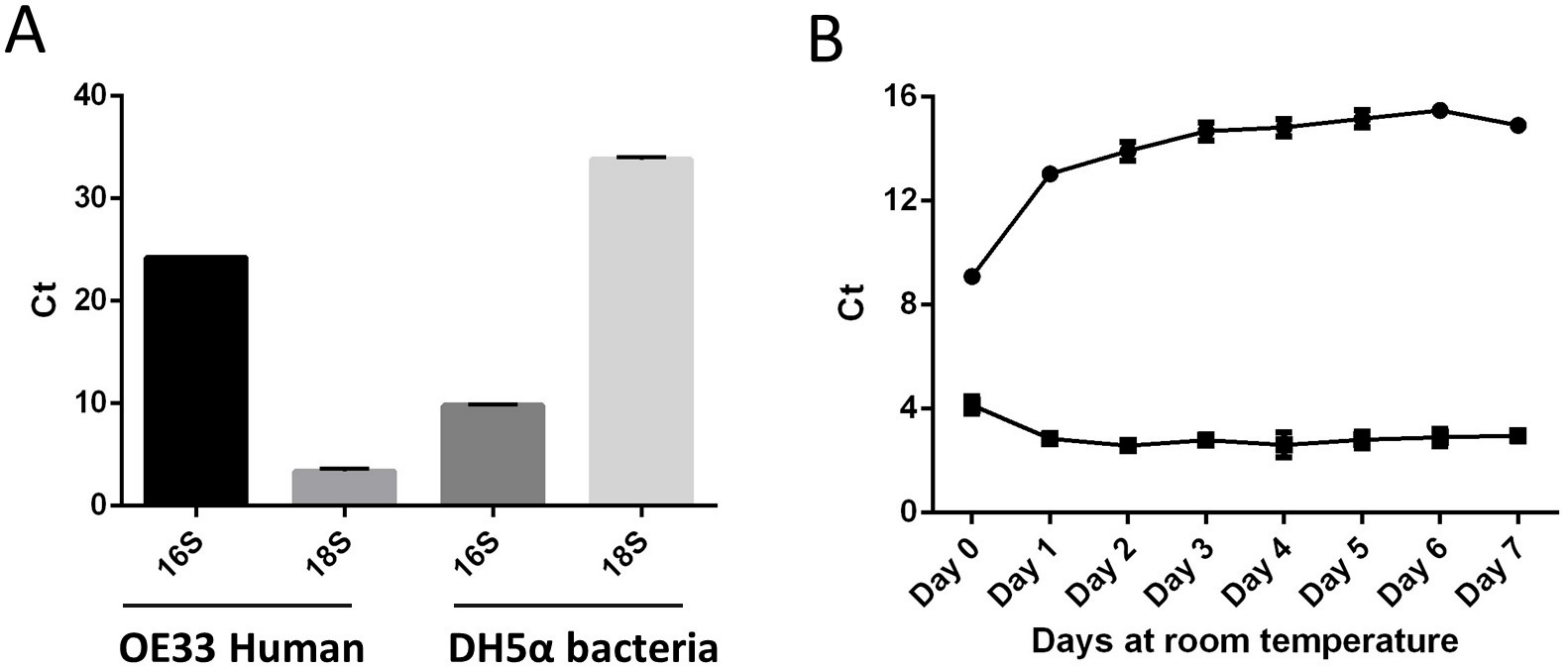
Effects of ambient storage length on salivary microbial growth. Primers for human 18S and bacterial 16S were tested on RNA from OE33 cells and DH5α bacteria respectively (n = 6). (**A**) Three pools of saliva collected in RNA*later* were created from nine volunteers and RT-PCR was performed using human 18S and bacterial 16S primers to give raw Ct scores that are inversely proportional to expression. (**B**) Bacterial 16S values are shown as squares and human 18S primers are shown as circles. A one-way ANOVA and Tukey test were performed, statistical significance between days can be found in S1 and S2 Tables. The error bars represent SD.

It was noted that salivary samples became increasingly cloudy in colour the longer they were stored at room temperature. The bacterial load in the samples was quantified using qPCR of bacterial 16S primers compared to human 18S primers (refer to S1 and S2 Tables for statistical data). The raw Ct scores showed that bacterial 16S level remained stationary over time, confirmed using a DH5α bacterial control (Fig 3A). The raw Ct values for human 18S showed higher Ct values at all days compared to baseline, with Ct values rising each day, in comparison to values for day 0 (p < 0.0005) (Fig 3B). For human primers OE33 oesophageal cells were used as a positive control. As Ct values are inversely proportional to expression suggests a decrease in human 18S the longer the saliva sample is stored at room temperature.

### Overnight fasting increases salivary nucleotide concentration

**Fig 4:**
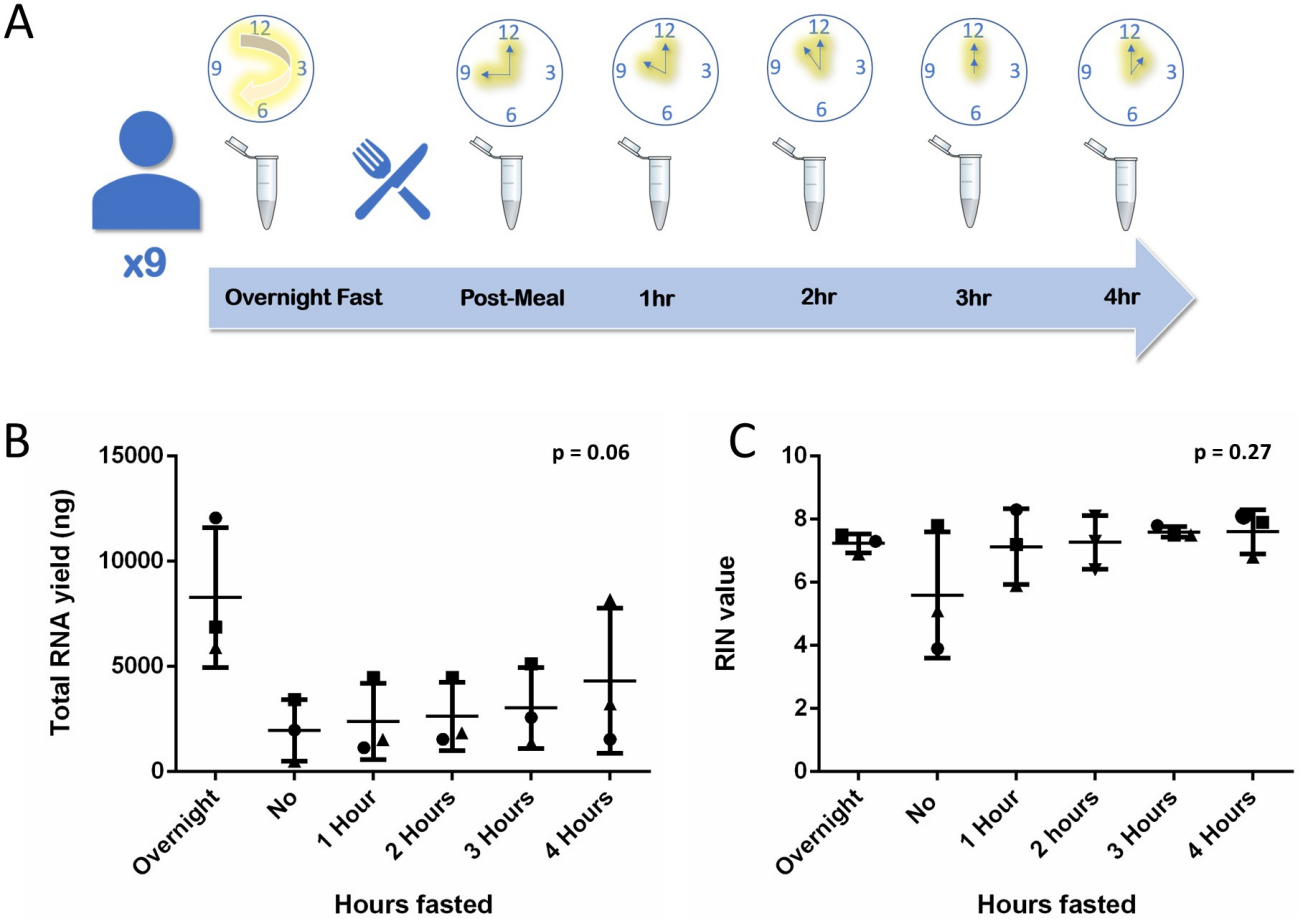
Effects of fasting window length on salivary RNA yield and quality. (**A**) Saliva was collected in RNA*later* from nine volunteers after overnight fasting, immediately after eating and then after fasting for 1–4 hours and pooled for each increment (n = 3). (**B & C)** RNA concentration (ng/μl) and RIN value were assessed. A one-way ANOVA and Tukey test were performed, with no significance found. The three different pools of samples are represented as circles, squares or triangles. The error bars represent SD.

Incrementally increasing fasting windows were used to investigate whether fasting has any effect on RNA concentration, yield or quality. RNA yield increased from 1970 μg ± 1458.2 immediately after eating to 8279.3 ng ± 3318.2 (p = 0.063) following an overnight fast. RNA concentrations increased from 49.25 μg/μl ± 36.46 immediately after eating to 206 ng/μl ± 82.96 (p = 0.063) following an overnight fast (Fig 4 and S2C Fig). This trend towards higher concentrations did not quite reach statistical significance when a post ANOVA TUKEY test, was applied (p = 0.06, Fig 4B). There was no change in RIN with increasing fasting length (p < 0.27), (Fig 4).

### Duration of freezing does not impact on nucleotide quality or quantity

**Fig 5:**
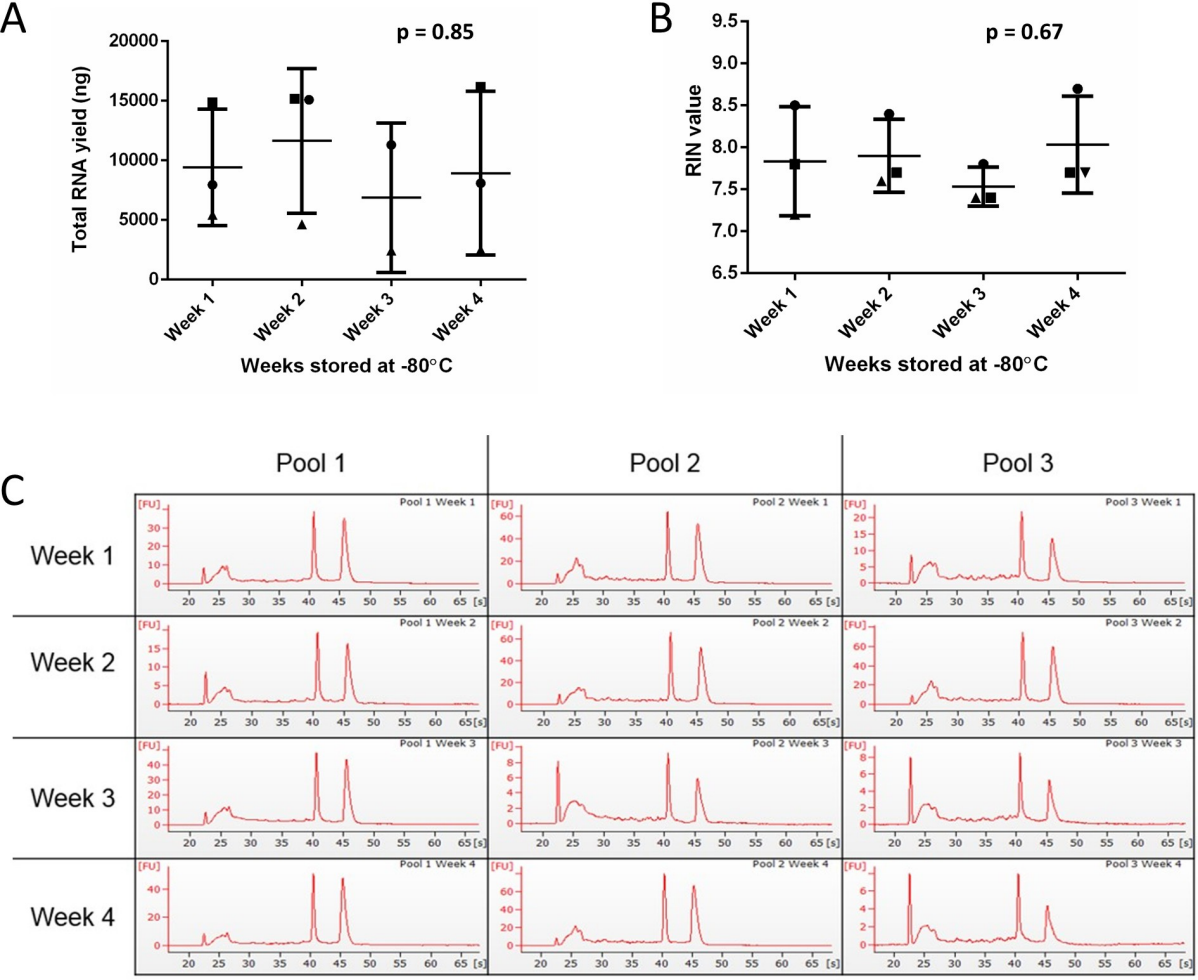
Effect of saliva storage length at -80 °C on RNA yield and concentration. Saliva was collected in RNA*later* from nine volunteers, pooled into three pools and aliquoted four times. An aliquot from each pool was extracted at the end of weeks 1, 2, 3, and 4. (**A & B)** RNA yield (ng) and RIN value were assessed for each pool. A one-way ANOVA was performed, followed by a Tukey test. The error bars represent SD. (**C**) Bioanalyzer traces for each pool at each timepoint are shown.

To investigate whether storing saliva for longer periods of time at -80 °C prior to processing had any effect on RNA yield, concentration or quality, the samples were frozen and extracted each week for four weeks. There are no statistically significant changes in either RNA yield, concentration or RIN between samples that were frozen for up to one month (Fig 5 and S2D Fig).

### Using the optimised method in clinical practice

**Fig 6:**
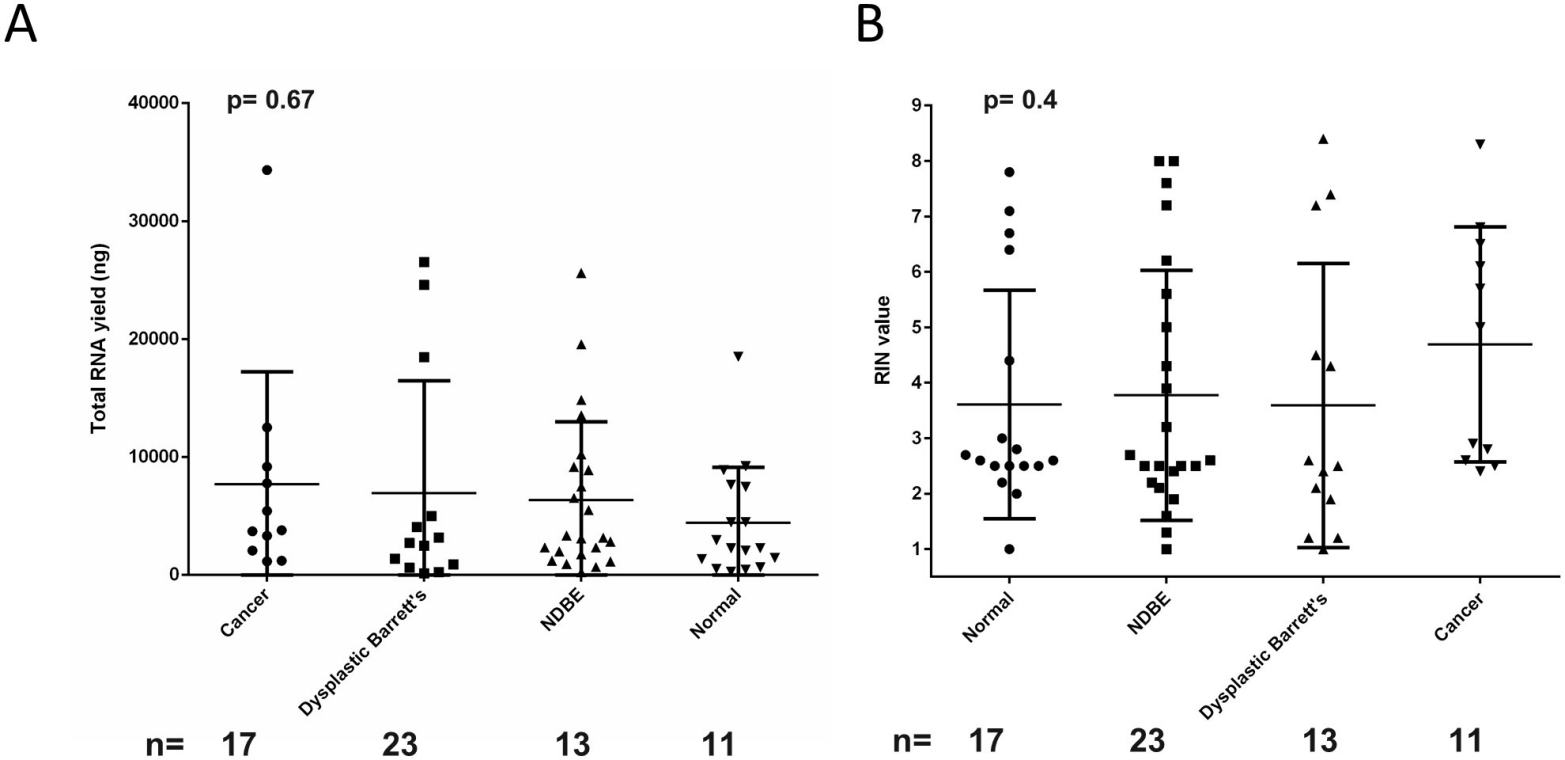
Clinical trial patient salivary RNA yield and quality. To assess whether the proposed collection method works within the clinic, the saliva from patients undergoing endoscopic diagnosis or treatments with diagnoses ranging from normal through non dysplastic Barrett’s oesophagus, through dysplasia to cancer was collected using the RNA*later* method. (**A & B)** The RNA yield (ng) and RIN value were assessed. A one-way ANOVA and Tukey test were performed. The error bars represent SD. IMC = intramucosal cancer, HGD = high grade dysplasia, LGD = low grade dysplasia, NDBE = non dysplastic Barrett’s oesophagus, NDSSBE = non dysplastic short segment Barrett’s oesophagus.

To demonstrate that this optimised collection method is clinically feasible, it was implemented during saliva sample collection from patients enrolled in clinical trials that were undergoing endoscopic intervention for Barrett’s Oesophagus and precancerous and cancerous conditions (n = 65). The mean RNA yield was 6307.1 ng (± 7324.2 ng), mean RIN was 3.9 (±2.23 ng/μl) and mean RNA concentration was 174.79 ng/μl (± 231.35 ng/μl) (Fig 6 and S2E Fig), with no statistical significance found between patients with different diagnoses.

## Discussion

One of the major challenges of working with saliva is the dilution of molecules compared to serum and the rapid degradation of analytes such as RNA. Currently there is no standardisation in saliva collection methods for human RNA analysis, and a previous study has recommended a method of RNA extraction that avoids the use of RNA stabilizers [27]. Studies do not commonly use RNA stabilisers in their saliva collection methods, instead employing collection over ice and ultra-low temperature storage, with RINs of approximately 2.5 from salivary RNA previously reported [1,25,3,4,26,27]. We have demonstrated that a major advantage of using preservative is a statistically significant increase in RNA concentration and quality with the addition of the RNA stabilizer RNA*later*, in comparison to saliva collected without preservative (Fig 1). Interestingly the saliva collected without preservative yielded higher quality RNA than saliva collected with DNA/RNA Shield, which we speculate may be due to the stringently controlled conditions under which the saliva collected without preservative was handled whilst on ice and promptly stored at -80 °C, aiding RNA preservation. This method of collection would not be clinically feasible however. Our collection method put the RNA*later* directly into the pre-prepared collection tube; ensuring saliva mixed with preservative almost immediately on collection, increasing the likelihood of it being effective. Pre-prepared tubes also work well within a busy clinical setting, where the use of laboratory chemicals is inappropriate. We have reported marked improvement in the median RINs of 7.0 (±0.3) for healthy volunteers and 3.9 (±2.2) for patient samples using this collection method. The median RIN achieved in the clinical setting vary from the RIN we report in our healthy volunteers due to the variable amounts of saliva we were able to collect from patients prior to their procedure caused by time-constraints and the general health of the patient. However 3.9 is an improvement on the RIN of 2.5 previously reported, despite the limitations in collection [1,25,3,4,26,27]. There were differences between the median age ranges of the healthy cohort and clinical cohort, due to the nature of the clinical trial. Both cohorts had a broad range of ages and therefore reflected the heterogenous nature of the general population. Saliva composition is influenced by factors such as health, oral hygiene and polypharmacy, as well as age, illustrating the variability which is difficult to control [30]. We have validated that this method works within the clinic where there is significant heterogeneity within a patient cohort and shown that the workflow is achievable in a clinical setting. Despite reports that cancer patients have increased RNase activity, there was no significant difference in RNA quality between different diagnoses, notably cancer and normal patients, promoting saliva as a viable medium for assessing biomarkers systemic disease including cancer [24].

Saliva has advantages as a matrix due to its ease of collection and accessibility, facilitating the possibility of samples being collected in the primary care setting, or even at home. Our data support the RNA*later* manufacturer’s instructions suggesting that samples can be left up to a week without compromising RNA quality, potentially enabling sample postage to a clinical laboratory (Fig 2A and 2B).

The oral microbiome is complex and over a period of days saliva stored at ambient temperature became cloudy in appearance and pungent, suggesting bacterial growth could be responsible for the increase in RNA quantity (Fig 2A) [17,19]. 16S rRNA amplicon analysis is the standard approach to microbial diversity investigations, and our data comparing bacterial 16S and human 18S shows an non-significant decrease (p < 0.5) in raw bacterial 16S Ct scores between days 0 and 1 which could be from an increase in bacterial RNA stabilisation by the RNA*later* [31]. However, bacterial 16S Ct scores stay consistent thereafter, suggesting no further bacterial DNA increase, aligning with the bacteriostatic nature of RNA*later*, whereby bacteria is prevented from growing but not necessarily killed [32]. However, over the same timeframe there is a statistically significant reduction in human 18S DNA in samples stored for 1–7 days in comparison to sample stored on day 0 at -80°C (p < 0.0005), suggesting RNA degradation. 16S amplicon analysis is specific for bacteria and does not rule out contamination from other organisms, such as fungi or yeast which make up the oral microbiome, which could explain the increase in RNA concentration over time [5]. If contamination of this kind is present, it could be contributing to the reduction in human 18S RNA seen in samples, in the absence of bacteria.

As saliva production and molecular content alters after eating food, previous studies have employed a fasting window of 1–1.5 hours prior to saliva collection [1,3]. Shorter fasting windows minimise patient discomfort. Our results suggest that the quality and yield of RNA is reduced with shorter fasting windows (Fig 4A and 4B). Although no statistical significance was found, our results suggest an overnight fasting window is optimal for RNA concentration and quality, possibly because salivary flow rates fall to near zero at night [16]. The notable decrease in RNA concentration (p = 0.063) (S2C Fig) in saliva collected immediately after eating suggests that the stimulation of saliva through the mechanisms associated with eating and drinking do reduce the abundance of RNA. We show that the RIN increases to a consistent level after 1 hour fasting and the yield steadily increases between 1–4 hours fasted. This raises the possibility that only a 1 hour fasting window is required to produce intact RNA, with subsequent hours fasting increasing the concentration. Our method can be tailored to the intended downstream application with two standardised fasting windows; a shorter one hour fast for high RINs or an overnight fast for higher yields. However, it may be the case that a larger cohort is needed to see significant effects, if any, of fasting and also storage temperature or length, on salivary analytes. Further work using larger numbers and perhaps longer and shorter fasting windows may allow for a more in-depth insight into applying this to saliva collection.

## Conclusion

Our results suggest that for future salivary studies unstimulated saliva is collected via passive drool using RNA*later* following an overnight fasting window to ensure high quality RNA-yields. Our results support the manufacturer’s guidelines that saliva should be stored atan ambient temperature for at least one day prior to storage.

We have described a robust and practical method for collecting high quality, high yield saliva that can be tailored for specific downstream applications. We have shown that this method can work routinely within a clinical setting, indicating it could be used across multiple centres. Once the bacterial growth has been investigated further this method could be tailored to primary care or at-home saliva collection, allowing for samples to be sent via post to a research laboratory setting. We recommend adoption of this standardised method for future salivary RNA studies.

## Supporting information

S1 Fig PCR primer specificity. Primer specificity was eastablished using melt curves and calibration slopes for 18S (A and C) and 16S (B and D) genes.
(TIF)

S2 Fig RNA concentration of samples under different testing conditions. Based on the volume of sample eluted and the concentration the yield was calculated for samples shown in Fig 1, comparing different preservatives with no preservative (A). Also for samples shown in Fig 2, comparing how many days at room temperature samples were stored at prior to freezing (B). Data is also shown for samples using the fasting experiment Fig 4 (C) and the length of time samples were stored at -80°C prior to extraction shown in Fig 5 (D).
(TIF)

S1 Table Statistical significance for 18S primer Ct values following qPCR analysis. * = p < 0.05, ** = p< 0.05, *** = p < 0.0005, Ns = Not Significant.
(TIF)

S2 Table Statistical significance for 16S primer Ct values following qPCR analysis. * = p < 0.05, ** = p< 0.05, *** = p < 0.0005, Ns = Not Significant.
(TIF)
