# Supplementary figures and images for: An optimised saliva collection method to produce high-yield, high-quality RNA for translational research
Source: PLoS One. 2020 Mar 9;15(3):e0229791. doi: 10.1371/journal.pone.0229791 (PMC7062242; doi:10.1371/journal.pone.0229791)

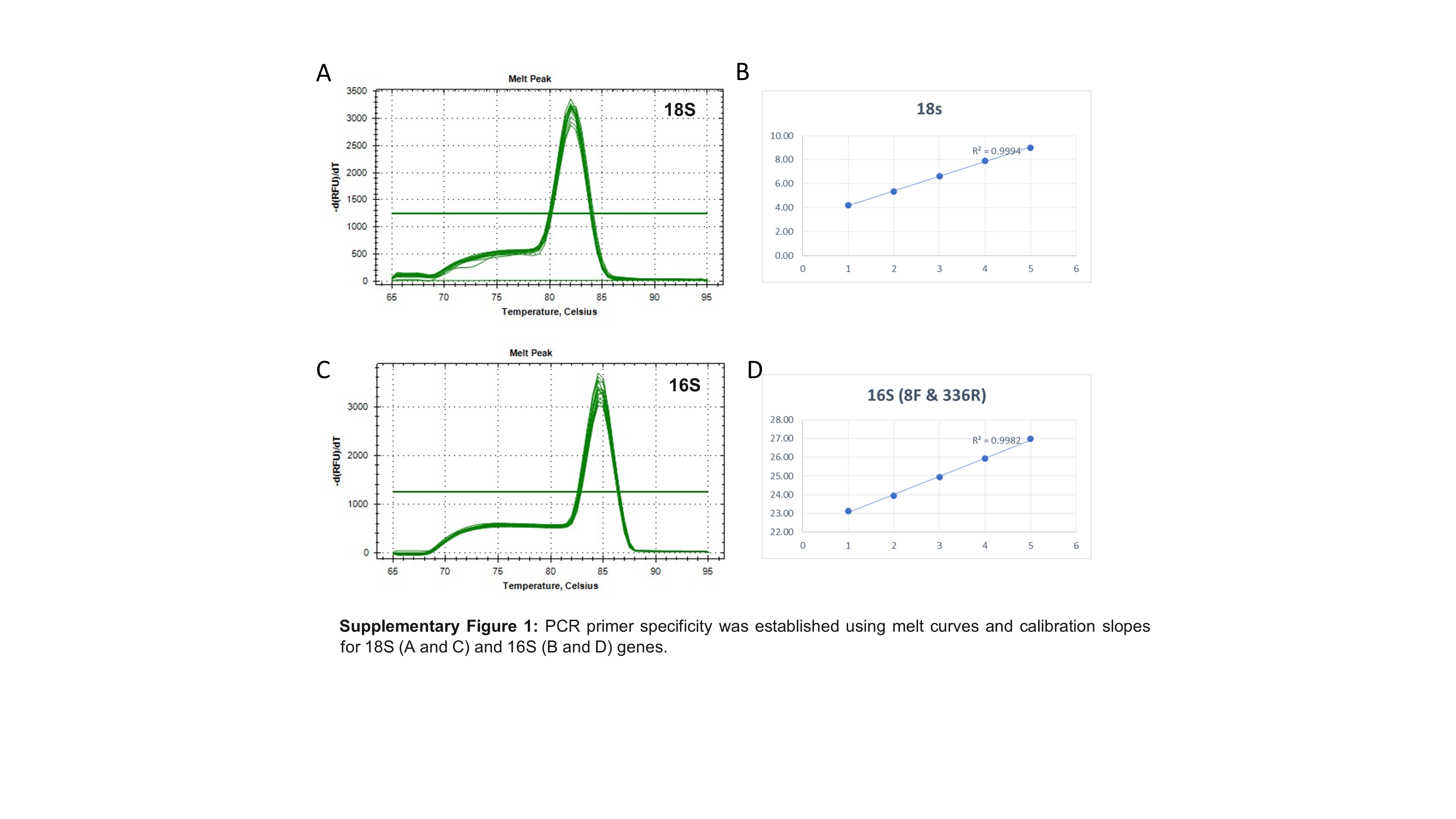

Supplement: S1 Fig — Primer specificity was eastablished using melt curves and calibration slopes for 18S (A and C) and 16S (B and D) genes. (TIF) [file pone.0229791.s001.tif]

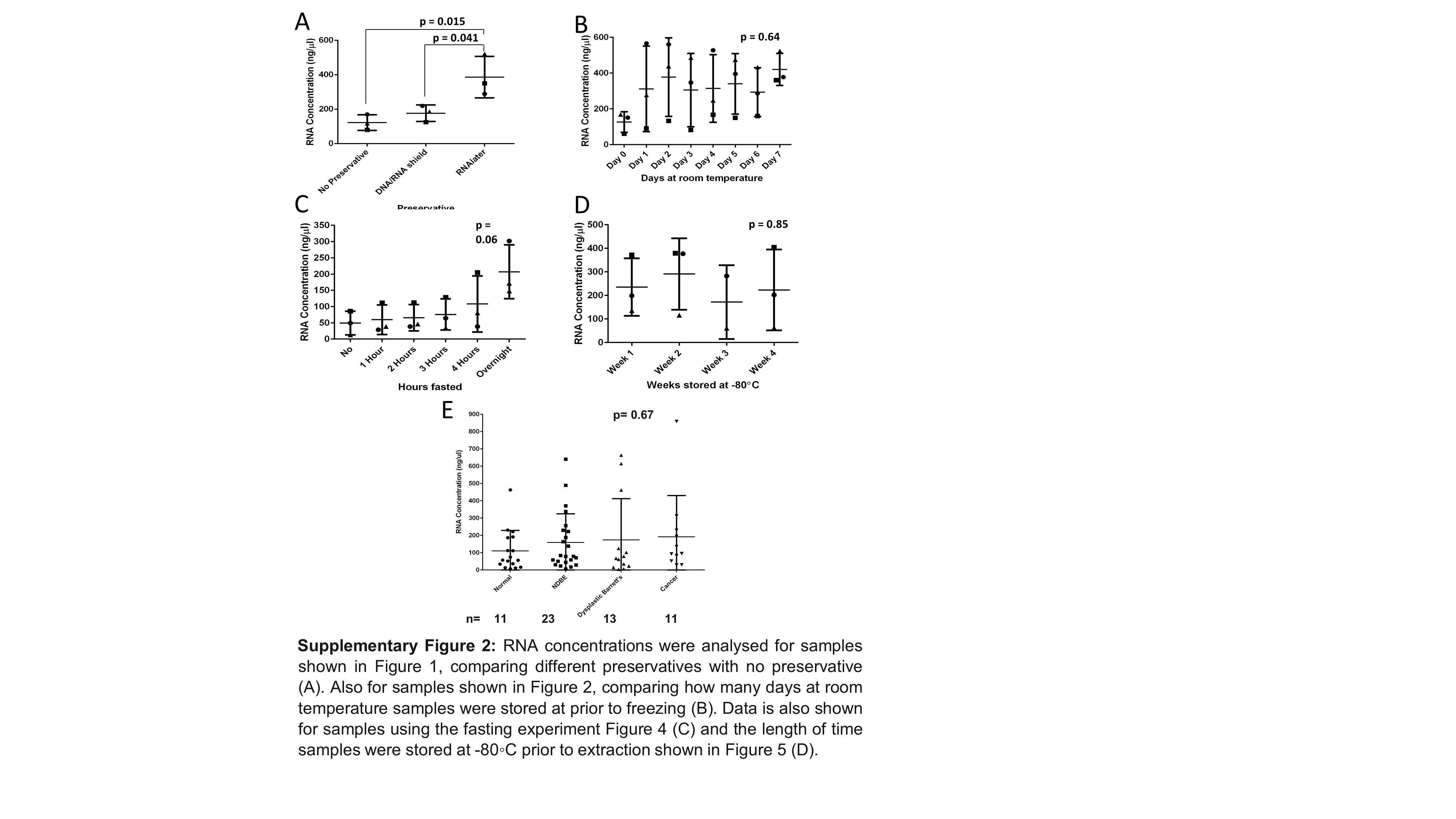

Supplement: S2 Fig — Based on the volume of sample eluted and the concentration the yield was calculated for samples shown in Fig 1, comparing different preservatives with no preservative (A). Also for samples shown in Fig 2, comparing how many days at room temperature samples were stored at prior to freezing (B). Data is also shown for samples using the fasting experiment Fig 4 (C) and the length of time samples were stored at -80°C prior to extraction shown in Fig 5 (D). (TIF) [file pone.0229791.s002.tif]

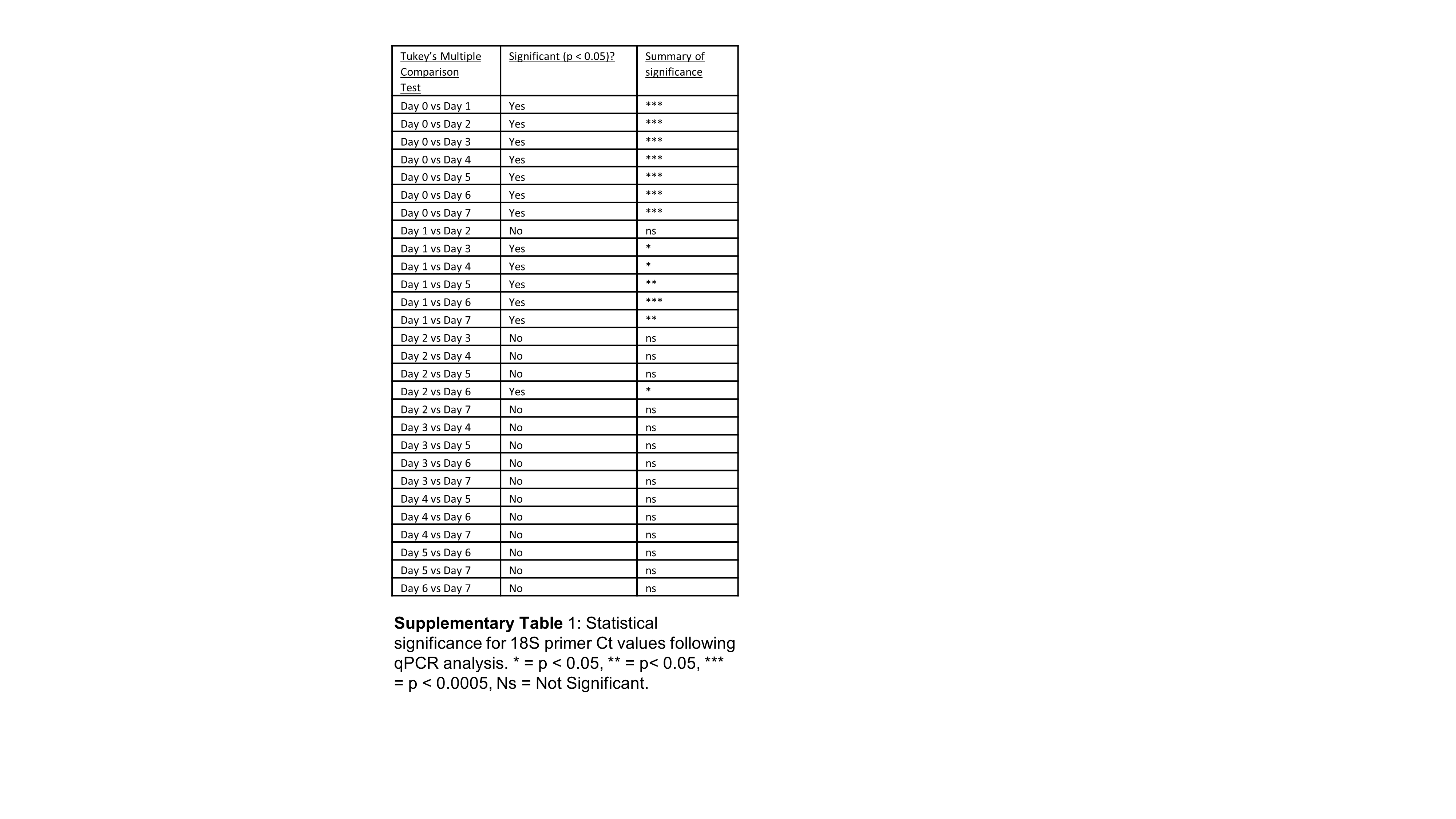

Supplement: S1 Table — * = p < 0.05, ** = p< 0.05, *** = p < 0.0005, Ns = Not Significant. (TIF) [file pone.0229791.s003.tif]

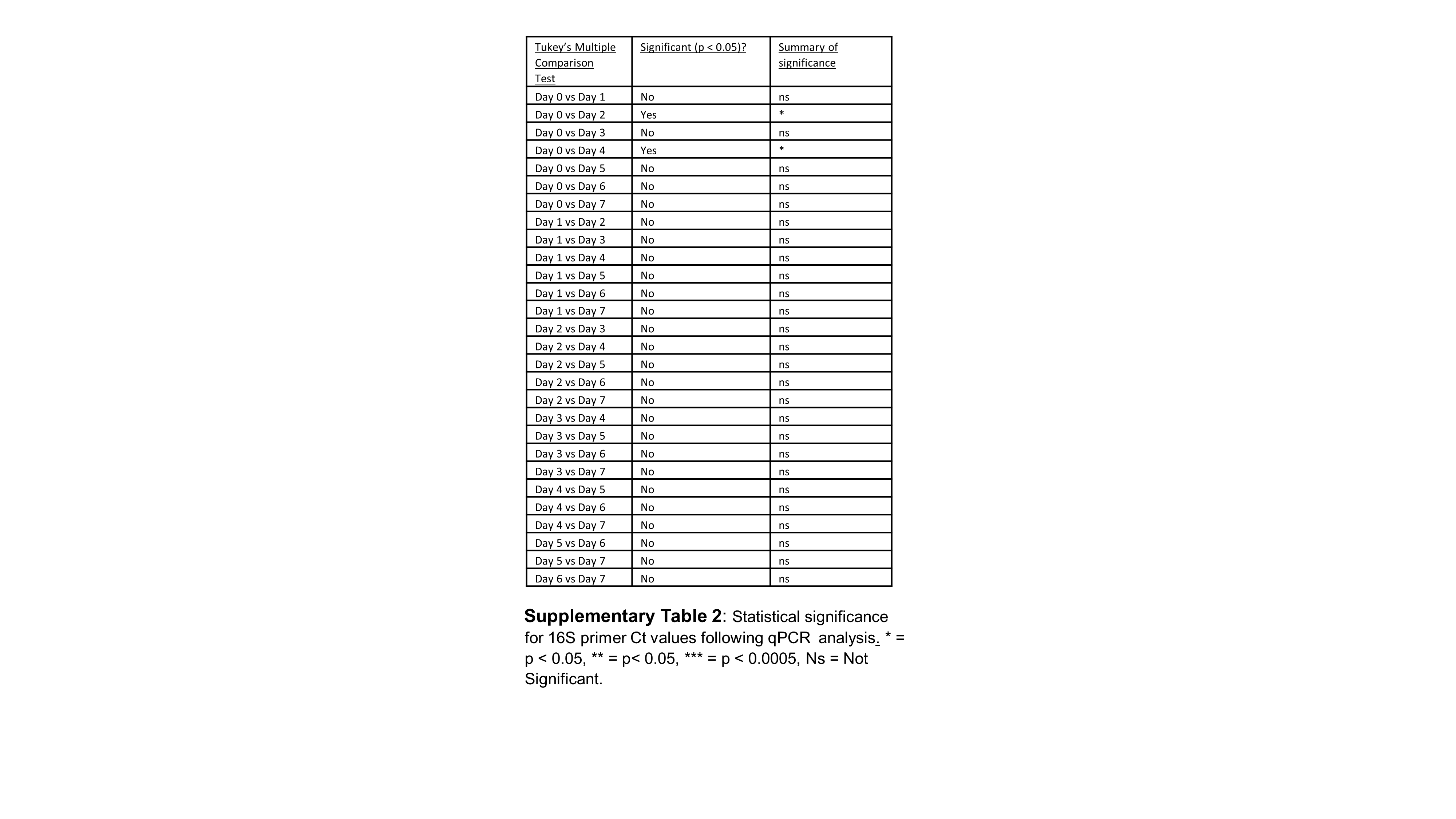

Supplement: S2 Table — * = p < 0.05, ** = p< 0.05, *** = p < 0.0005, Ns = Not Significant. (TIF) [file pone.0229791.s004.tif]
